# Re-evaluating the role of TPJ in attentional control: Contextual updating?^☆^

**DOI:** 10.1016/j.neubiorev.2013.08.010

**Published:** 2013-12

**Authors:** Joy J. Geng, Simone Vossel

**Affiliations:** a Center for Mind and Brain and Department of Psychology, University of California Davis, 267 Cousteau Pl., Davis, CA, USA; b Wellcome Trust Centre for Neuroimaging, University College London, WC1N 3BG London, UK; c Cognitive Neuroscience, Institute of Neuroscience & Medicine (INM-3), Research Centre Juelich, 52425 Juelich, Germany

**Keywords:** Temporo-parietal junction, Attention, Ventral attentional control network, Theory of mind, Inferior parietal cortex

## Abstract

The right temporo-parietal junction (TPJ) is widely considered as part of a network that reorients attention to task-relevant, but currently unattended stimuli (Corbetta and Shulman, 2002). Despite the prevalence of this theory in cognitive neuroscience, there is little direct evidence for the principal hypothesis that TPJ sends an early reorientation signal that “circuit breaks” attentional processing in regions of the dorsal attentional network (e.g., the frontal eye fields) or is completely right lateralized during attentional processing. In this review, we examine both functional neuroimaging work on TPJ in the attentional literature as well as anatomical findings. We first critically evaluate the idea that TPJ reorients attention and is right lateralized; we then suggest that TPJ signals might rather reflect post-perceptual processes involved in contextual updating and adjustments of top-down expectations; and then finally discuss how these ideas relate to the electrophysiological (P300) literature, and to TPJ findings in other cognitive and social domains. We conclude that while much work is needed to define the computational functions of regions encapsulated as TPJ, there is now substantial evidence that it is not specialized for stimulus-driven attentional reorienting.

## Highlights

• We examine the function of the temporo-parietal junction (TJP) in the human brain.
• The function of TPJ is critically evaluated using data from cognitive neuroscience.
• We conclude TPJ function in many domains is well described by contextual updating.

## 1 Introduction

The right temporo-parietal junction (TPJ) is widely considered to be a critical part of a right-lateralized ventral attentional control network that reorients attention toward the appearance of unexpected, but task-relevant objects (Corbetta and Shulman, 2002; Corbetta et al., 2008; Shomstein, 2012). This view is almost ubiquitous within the field of attention and has been heavily drawn upon by other psychological domains (e.g., Cabeza et al., 2008; Ciaramelli et al., 2008; Mitchell, 2008; Frank and Sabatinelli, 2012). However, despite many demonstrations that TPJ responds to stimuli that are unexpected and task-relevant, there is little direct evidence for two specific aspects of the model: First, that TPJ activity triggers the reorientation of attention (in space, time, or by feature); and second, that TPJ function in attentional control is strictly right-lateralized.

**Fig. 1:**
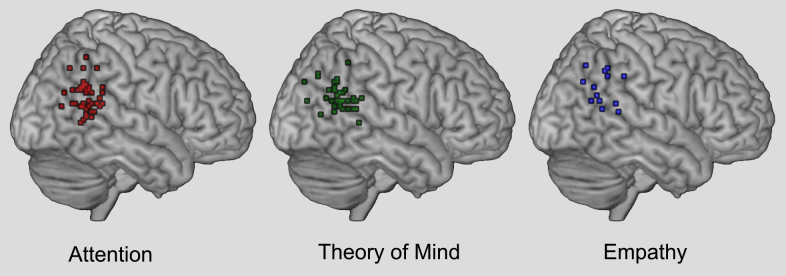
Peak voxel coordinates for attention, theory of mind, and empathy. Coordinates were derived from the meta-analysis by Decety and Lamm (2007a,b). Additional data points from more recent studies have also been added to the visualization (see Table 1 for references of studies included). Images of the peak voxel coordinates in MNI space were created using GingerALE (www.brainmap.org) and are depicted on the MRIcroN (http://www.mccauslandcenter.sc.edu/mricro/mricron/) template brain.

**Fig. 2:**
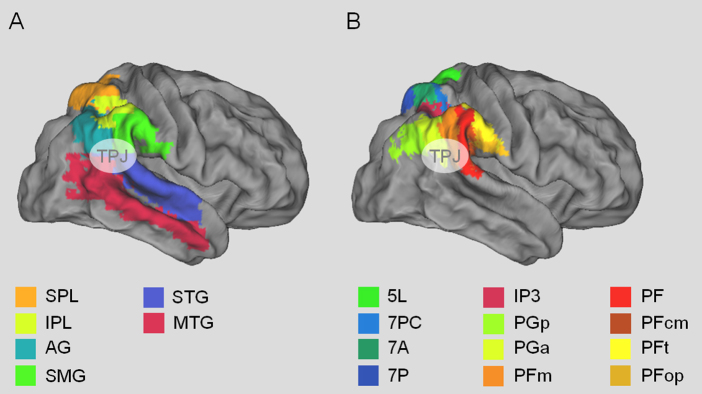
Illustration of the anatomical location of the parietal cortex from the Automatic Anatomical Labeling (AAL) atlas (Tzourio-Mazoyer et al., 2002) (A) and the cytoarchitectonic parietal maps of the Juelich atlas (Eickhoff et al., 2005) (B). The maps are depicted on the flattened brain surface of the PALS atlas as implemented in Caret 5.65 (Van Essen, 2005). SPL: superior parietal lobe, IPL: inferior parietal lobe, AG: angular gyrus, SMG: supramarginal gyrus, STG: superior temporal gyrus, MTG: middle temporal gyrus.

Understanding TPJ is important not only because attention is a core cognitive function that contributes to many other domains of information processing, but also because TPJ has been identified as a key structure in seemingly unrelated domains (e.g., memory, body representation, theory-of-mind). In all of these domains, the attentional account of TPJ has played a critical role to either explain function or fuel debate over functional specialization (Decety and Lamm, 2007a; Hein and Knight, 2008; Mitchell, 2008; Perner and Aichhorn, 2008; Greene et al., 2009; Scholz et al., 2009; Young et al., 2010). In both cases, these cross-domain exchanges assume that the responses within TPJ are fundamentally dedicated to computations in either attentional reorienting or another domain. The argument follows that if the regions (or neurons) activated by both cognitive domains (or tasks) are the same, then one function must explain the other; if they are physically separate, then they can be independent modules (see Table 1 and Fig. 1 for peak coordinates of findings attributed to TPJ from different domains; see Fig. 2 for anatomical landmarks). However, a third possibility is that there is a domain-general computation in TPJ that is neither specific for attentional reorienting nor for a single other cognitive process such as ToM, but nevertheless critically underlies all of them (see also, Seghier, 2013).

The purpose of this review is to critically evaluate two core assumptions about the role of TPJ in attentional control in order to highlight the need to reconsider theories of TPJ function. As an alternative account, we then propose the “contextual updating” hypothesis in which the function of TPJ is to update internal models of the current behavioral context for the purpose of generating appropriate actions; contextual updating is therefore particularly important when unexpected stimuli occur. A similar idea has long existed within the event-related potentials (ERP) literature with respect to the P300 component, which is thought to have a number of neural sources including TPJ (Kutas et al., 1977; Donchin, 1981; Knight et al., 1989; Verleger et al., 2005).

While the contextual updating hypothesis implies an unitary account for TPJ function, we readily acknowledge that it is likely that there are multiple specialized sub-regions in “TPJ” (Scholz et al., 2009). However, given the number of different cognitive and emotional processes that have been identified in the larger TPJ region (Decety and Lamm, 2007b; Nelson et al., 2010; Jakobs et al., 2012; Langner and Eickhoff, 2013; Seghier, 2013) and uncertainty regarding the anatomical separation of different functions (Mitchell, 2008; Scholz et al., 2009; Hutchinson et al., 2012) (compare Figs. 1 and 2), we believe that it is currently still useful to identify common computational principles across domains rather than to fragment the region into separate cognitive modules (Fodor, 1983). After all, we do not know what the dividing principles should be and they may very well violate the topical and task-based divisions we currently rely on to divide fields of study. Our approach is in line with other recent theories that provide integrative explanations for why TPJ may be found in multiple domains of study (Graziano and Kastner, 2011; Cabeza et al., 2012; Frank and Sabatinelli, 2012; Seghier, 2013).

The review is structured in five sections: the first reviews the evidence for the role of TPJ in attention based on human functional magnetic resonance imaging (fMRI) and neuropsychology; the second reconsiders the evidence for two commonly accepted characteristics of TPJ in attention; the third discusses the contextual updating hypothesis for TPJ with respect to studies of attention; the fourth section evaluates (necessarily in brief) the generalizability of the contextual updating hypothesis to other psychological domains; finally, in the fifth section, we discuss recent anatomical work that contributes importantly to the anatomical definitions for the region of cortex broadly referred to as TPJ.

## 2 TPJ in attentional selection

### 2.1 Functional imaging

Perhaps the most prevalent theory of TPJ function, particularly in the right hemisphere, comes from Maurizio Corbetta and colleagues (Corbetta and Shulman, 2002; Corbetta et al., 2008). In this theory, TPJ is part of a “ventral attentional control network” that also includes the inferior frontal gyrus and middle frontal gyrus. This ventral network is thought to be responsible for the reorientation of attention to behaviorally relevant, but currently unattended stimuli (note that the reorientation need not be spatial in nature) (Downar et al., 2001; Corbetta and Shulman, 2002). Stimulus-driven changes in attentional focus are frequently referred to as “bottom-up” reorientation and can be understood in contrast to “top-down” mechanisms that control voluntary attentional selection (for review see Shomstein, 2012). Early evidence for TPJ in reorienting attention came from variations of the Posner task in which spatial cues indicate the position of behaviorally relevant targets with a given probability (Posner et al., 1980). Greater right hemispheric TPJ activation occurred in response to invalidly cued targets (Rosen et al., 1999; Arrington et al., 2000; Corbetta et al., 2000; Kincade et al., 2005; Hahn et al., 2006; Vossel et al., 2006; Indovina and Macaluso, 2007; Doricchi et al., 2010; Natale et al., 2010). For example, Vossel et al. (2006) found greater TPJ activation in response to invalid targets and moreover, invalid target activation was greater when the cue validity was 90% as compared to only 60% and that this corresponded with the size of the behavioral validity effects (i.e., the difference in RT between invalid and valid targets). This suggested that activation in right TPJ and the right frontal cortex was commensurate with the strength of the violation of expectations. Importantly, TPJ responses are not due to “bottom-up” sensory salience as task-irrelevant salient stimuli do not activate TPJ, but rather FEF and IPS (Kincade et al., 2005; Indovina and Macaluso, 2007; Geng and Mangun, 2009).

In addition to spatially “invalid” targets, TPJ is also sensitive to relevance defined by other stimulus dimensions such as: target-similarity (Serences et al., 2005; Natale et al., 2010; Chang et al., 2012), cues that indicate a need to shift spatial attention (Shulman et al., 2010; Geng and Mangun, 2011), as well as “surprising” stimuli that may reorient attention, albeit not in the spatial dimension (Asplund et al., 2010). Although these stimuli are superficially dissimilar to a target in an invalidly cued spatial location, they are all external stimuli that trigger a shift in attention because they have features that are potentially relevant for a task-based decisions. Together, such results led to the idea that TPJ and the ventral attentional network are suppressed during “top-down” guided attentional processes, but are activated in order to “circuit-break” the voluntary attentional control system to reorient attention to unexpected, but relevant information (Corbetta and Shulman, 2002; Shulman et al., 2007).

### 2.2 Spatial neglect

Support for the notion that right TPJ plays a crucial role in reorienting attention to unexpected sensory stimuli comes from spatial neglect. Spatial neglect is a neuropsychological phenomenon that is defined by behavioral symptoms of a deficit in spontaneously reorienting attention to stimulus information in the contralesional visual field. Spatial neglect cannot be attributed to either sensory or motor defects alone (Kinsbourne, 1977; Mesulam, 1999; Halligan et al., 2003; Heilman et al., 2012) and is associated with damage primarily to the right inferior parietal cortex (Posner et al., 1984; Vallar and Perani, 1986; Karnath et al., 2003; Mort et al., 2003; Behrmann et al., 2004; Thiebaut de Schotten et al., 2005; Shomstein, 2012). For example, Mort et al. (2003) found that the region of greatest overlap for patients with neglect following middle cerebral artery (MCA) territory stroke was in the white matter just beneath the rostroventral portion of the right angular gyrus. Moreover, the lack of damage to this region was a good predictor of patients without neglect, suggesting that the inferior parietal cortex makes a critical contribution to stimulus-driven attentional orienting. Others, however, have argued that that the critical brain correlate of neglect is the right superior temporal sulcus (STS) (Karnath, 2001; Karnath et al., 2003; Behrmann et al., 2004; Ellison et al., 2004), lesion volume, or disruption within a network of multiple regions (Mesulam, 1990; Maguire and Ogden, 2002; Peers et al., 2005; Vossel et al., 2011; Molenberghs et al., 2012). TPJ damage, including portions of the inferior parietal lobe and STS, has recently also been linked to anosognosia in neglect when visuospatial deficits are controlled for, and perceptual awareness more generally (Beauchamp et al., 2012; Vossel et al., 2012b). Going further, a recent theory hypothesizes that consciousness is a meta-process that extracts information from our own attentional states of which potentially separate parietal and temporal portions of the TPJ are critical (Graziano and Kastner, 2011). Taken together, the work on spatial neglect suggests that TPJ in the right-hemisphere encompasses areas that are critical for stimulus-driven attentional control and awareness.

## 3 Re-evaluation of two characteristics of TPJ

While the neuroimaging and neuropsychological data converge in support of the Corbetta et al. (2000, 2008) model of a right-hemispheric dominant ventral attentional control network that reorients attention by communicating with the dorsal network via frontal regions, there is also evidence that this model is unlikely to be correct in its entirety. Some of these concerns were raised originally by Corbetta and colleagues (e.g., Corbetta et al., 2008), while others have emerged more recently. The first issue involves the timing of activity in TPJ vs. nodes of the dorsal network such as FEF. The second involves the asymmetric characterization of contributions from left and right TPJ to attentional control processes. We review each of these in turn.

### 3.1 Evidence against a specific role for TPJ in the reorienting component of attention

If TPJ is to send a fast-latency “interrupt” signal to the dorsal network that reorients attention, it logically follows that TPJ must generate an earlier output signal in response to sensory inputs than the dorsal system regions. However, the evidence from electrophysiology and TMS suggests that FEF actually responds earlier, not later, to sensory information than TPJ. For example, the latency of stimulus-evoked responses in FEF range from 50 to 138 ms (Mohler et al., 1973; Thompson et al., 1996) and when FEF TMS is applied with temporal precision using double pulse TMS time-locked to stimulus onset, it affects stimulus-driven attentional orienting 30–90 ms after stimulus onset (O'Shea et al., 2004; Neggers et al., 2007; Bardi et al., 2012). In addition, covert attentional capture effects appear to be even earlier than those signals associated with saccadic eye-movements (Juan et al., 2004, 2008). In contrast, TMS of TPJ has a later effect around 150 and 250 ms after stimulus appearance (Meister et al., 2006).

The precise timing of repetitive TMS is harder to evaluate, but has been reported to impair visuospatial attention tasks when applied over the ventral posterior parietal cortex for 500 ms or longer (Ellison et al., 2004; Schindler et al., 2008; Chang et al., 2012). Additionally, human neurophysiological data on a perceptual phosphene induction task found TPJ gamma activity began 100 ms after direct electrical stimulation of occipital cortex (*not* stimulus onset) and lasted 200 ms with an approximate peak at 200 ms (Beauchamp et al., 2012). However, the fact that temporally late TMS of TPJ impacts performance does not preclude the possibility that TPJ also sends an earlier signal. One possibility is that TPJ is involved in early and late phases of information processing (Chambers et al., 2004a). Using TMS, Chambers et al. (2004) found that a post-cue disruption of the right angular gyrus at 90–120 and 210–240 ms reduced accuracy for invalid targets in a cued attention task. Interestingly, they suggest that the early signal may reflect attentional disengagement (not reorienting). Thus, while TPJ may be involved in recurrent interactions with dorsal attentional control regions such as FEF, the anterior cingulate, and other regions of prefrontal cortex (Mesulam, 1990; DiQuattro and Geng, 2011; Geng and Mangun, 2011; Ristic and Giesbrecht, 2011; Vossel et al., 2012a; DiQuattro et al., 2013), the evidence does not suggest that there is an early signal from TPJ that causally reorients attention to a new location or feature.

ERP data recorded from scalp EEG also provide some evidence that the signal associated with TPJ is likely to occur later than that of attentional orienting signals originating in FEF and other dorsal network regions. The ERP most associated with TPJ is the P3b subcomponent of the P300 (Sutton et al., 1965a; Menon et al., 1997; Linden et al., 1999a; Soltani and Knight, 2000; Polich, 2003, 2007). The P300 is subdivided into the P3a, which has a frontal distribution and occurs in response to novelty and the P3b, which has a more posterior scalp distribution and occurs in response to targets. Although both the P3a and P3b likely have multiple neural sources, TPJ (particularly in the supramarginal gyrus) appears to be the most prominent source for the P3b, which usually occurs 300–500 ms after stimulus onset (Menon et al., 1997; Linden et al., 1999b; Soltani and Knight, 2000; Bledowski et al., 2004a,b).

The P300 literature suggests that its neural generators encode the post-perceptual stimulus category as defined by the current task, and that this occurs relatively late after stimulus onset (Sutton et al., 1965b; Kutas et al., 1977; Johnson and Donchin, 1980). This is in contrast to the earlier timing of ERP components such as the N2pc and Pd that signal the shift of attention and the suppression of target-similar distracters, respectively (Luck and Ford, 1998; Woodman and Luck, 1999; Hickey et al., 2009; Sawaki et al., 2012). One long-standing theory is that the P300 reflects “contextual updating”, which involves the modification of one's internal model of the environment (e.g., task-based expectancies) based on an external stimulus (Donchin, 1981; Donchin and Coles, 1988; Polich, 2007). A related hypothesis is that the context updating refers specifically to information in working memory schemas (Vogel et al., 1998; Soltani and Knight, 2000; Luck, 2005; Polich, 2007). We further explore the relationship between contextual updating and existing findings in TPJ in Section 4.

In sum, the electrophysiological, TMS and ERP data are not consistent with the idea that TPJ sends an early reorientation signal to FEF that then shifts attention to task-relevant information (e.g., DiQuattro et al., 2013). Although it may be that TPJ plays an important role in attentional control and interacts recurrently with dorsal regions such as FEF (Ellison et al., 2004; Polich, 2007), the data suggest that the role of TPJ is not specifically for early stimulus-driven attentional reorienting, but involves integrating internal representations of the task context with stimulus and response expectations.

### 3.2 Hemispheric specialization: left and right TPJ

As noted above, neuropsychological work on attention found that the behavioral deficits in spatial attention were asymmetrical (e.g., in spatial neglect) following selective damage to each hemisphere (Ringman et al., 2004; Vallar and Perani, 1986; Vallar, 1993). Those findings led to a number of different theories for why such asymmetries occurred. One prominent model hypothesizes that the right hemisphere controls shifts of attention to both sides of space, but that the left hemisphere only controls shifts to the right (Mesulam, 1981). Another, the opponent-processes model, postulates that each hemisphere controls contralateral shifts by inhibiting the other hemisphere, but that the orienting signal is stronger in the left hemisphere. Thus, damage to the right releases the strong orienting response in the left hemisphere to the right side of space, but damage to the left has only a modest effect on right hemisphere orienting to the left side of space (Kinsbourne, 1977, although see Eshel et al., 2010).

A more recent model for neglect by Corbetta and Shulman (2011) suggests that the spatial asymmetries are due to impaired interactions between the ventral and dorsal attentional networks; they postulate that the non-spatial functions of the ventral attentional network, in particular TPJ, cause the interactions between the left and right hemisphere nodes of the dorsal network to become imbalanced. This model is closely linked to the idea that the ventral attentional network is right-lateralized, but that the dorsal network is bilateral. Consistent with this model, differences between the right and left superior longitudinal fasiculus III connecting BA40 to prefrontal regions of the dorsal attentional network have been identified and related to measurements of attentional selection (Schmahmann et al., 2007; Thiebaut de Schotten et al., 2011).

There appear to be clear hemispheric differences in attentional control, but there is also increasing evidence that the left TPJ plays an important role in control processes related to attention. For example, many studies in which right TPJ is found in response to task-relevant stimuli also report left lateralized activation in similar conditions, although the left hemisphere activation is rarely investigated in detail (Downar et al., 2000; Kincade et al., 2005; Serences et al., 2005; Anticevic et al., 2010; Geng and Mangun, 2011). A notable exception is a paper by Doricchi et al. (2010) in which both left and right TPJ activation were examined in a spatial cueing paradigm. Although the contrast between invalid minus valid targets produced the commonly reported right TPJ activation, they found upon closer inspection that left TPJ responded to both invalid and valid targets. This led them to hypothesize that left TPJ encodes targets that both match and that mismatch expectations whereas right TPJ only encodes mismatches. Such a pattern would lead to the cancelation of left TPJ in statistical maps based on the contrast of invalid minus valid targets (Doricchi et al., 2010). This hypothesis is consistent with bilateral activations in “oddball” target detection tasks (Menon et al., 1997; Linden et al., 1999b).

The left TPJ is also frequently activated in studies of memory (Cabeza et al., 2008; Hutchinson et al., 2009). This has led to an influential theory of episodic memory retrieval in which the posterior parietal cortex and TPJ specifically, encodes attentional capture by contents of memory that are task-relevant and match the current retrieval cue (Cabeza et al., 2008; Ciaramelli et al., 2008). The left-lateralization may be due to hemispheric specializations between memory vs. perceptual processes, or the fact that memory studies frequently use verbal stimuli whereas standard visual attention tasks use only perceptual stimuli (Hutchinson et al., 2009; Weidner et al., 2009; Ravizza et al., 2010; DiQuattro and Geng, 2011; Cabeza et al., 2012). Studies of patients with parietal damage suggest that the left posterior parietal cortex may be involved in the automatic awareness of retrieved information (Berryhill et al., 2007; Davidson et al., 2008), which is maybe more analogous to the idea that the left TPJ encodes “matches” between internal templates and external stimuli (Doricchi et al., 2010). Furthermore, connectivity analyses using RTPJ as a seed region often results in activation of the homologous left hemisphere region suggesting that TPJ in the two hemispheres are highly interconnected (Mooshagian et al., 2008; Geng and Mangun, 2011; Jakobs et al., 2012). Thus, while it may be that left and right TPJ have specialized functional roles, future research is required to delineate the functional relationship between the two hemispheres.

## 4 Contextual updating as a framework for role of TPJ in attentional control

In the previous section we reviewed evidence against the idea that TPJ provides an early stimulus-driven signal that triggers attentional reorienting. However, the existing data also clearly indicate that TPJ is engaged in processing task-relevant stimuli, particularly when they are unexpected. Here we suggest that the observed effects in TPJ can be understood in terms of “contextual updating” (see above) (Sutton et al., 1965b; Kutas et al., 1977; Johnson and Donchin, 1980; Donchin, 1981; Donchin and Coles, 1988; Verleger, 1988; Soltani and Knight, 2000; Verleger et al., 2005; Polich, 2007). Although there is an unresolved debate within the ERP literature regarding the exact context representations reflected by the P3a and P3b (Donchin and Coles, 1988; Verleger, 1988), we believe the contextual updating hypothesis provides a fitting framework for TPJ function (Langner and Eickhoff, 2013; Seghier, 2013). We therefore suggest that a candidate computation for TPJ might be one that updates an internal model of the current environmental context based on new sensory information; we hypothesize that the update initiates a task-appropriate (covert or overt) action and a change in expectations about upcoming events (Donchin, 1981; Verleger, 1988; Downar et al., 2000, 2002; Verleger et al., 2005; Astafiev et al., 2006; Husain and Nachev, 2007; Decety and Lamm, 2007a; Corbetta et al., 2008; Desmurget and Sirigu, 2009; Eickhoff et al., 2011; Geng and Mangun, 2011; Graziano and Kastner, 2011; Langner and Eickhoff, 2013; Seghier, 2013).

The contextual updating hypothesis is consistent with extant findings of TPJ activation in standard attentional tasks. For example, invalid targets in the Posner paradigm are infrequent and violate expectations and therefore produce an update to stimulus-response mappings of what action to take and what to expect in the future. Similarly, a valid target would also be predicted to activate TPJ, albeit to a lesser degree, because it signals the need to make a context-appropriate response based on the new occurrence of a target stimulus, but there is no violation of expectations (Kincade et al., 2005; Astafiev et al., 2006; Vossel et al., 2006, 2009; Doricchi et al., 2010).

The contextual updating hypothesis provides similar explanations for why target-colored distracters produce TPJ activation (Serences et al., 2005; DiQuattro et al., 2013). The appearance of a target-colored object in the wrong location violates expectations of what is to happen and requires an update to the appropriate response: the target-present response must be withheld and possibly a “non-target” response be made (depending on the task). Also similar to the spatial cueing results, targets in feature-based attention tasks would also be expected to produce significant activations in TPJ as they indicate the need to make a context-appropriate response, albeit an expected one. Mismatches to expectations produce the largest responses because they represent the most significant updates to the internal model, but matches may also be taken as evidence for the existing mental model (for similar ideas see also, Eickhoff et al., 2011; Seghier, 2013). Likewise, the appearance of a cue, or any stimulus, that requires a change in attention to anticipate task-relevant information will be followed by a model update of where relevant stimuli are likely to appear and what responses are required. Many studies that do not clearly require a reorientation of attention, but nevertheless do produce TPJ activation contain stimuli that produce such a contextual update to immediate actions or future expectations (Downar et al., 2000, 2001; Kincade et al., 2005; Shulman et al., 2009; Weidner et al., 2009; DiQuattro and Geng, 2011; Geng and Mangun, 2011; Philipp et al., 2012).

The contextual updating hypothesis also fits in well with the overall goal of organisms to establish predictive expectancies that facilitate behavioral actions (Rescorla and Wagner, 1972; Sutton et al., 1998; Behrens et al., 2007; Besle et al., 2011; Eickhoff et al., 2011; Friston, 2012). We do not suggest that TPJ is itself a module for predictive coding (Huettel et al., 2002; Summerfield et al., 2006; Shulman et al., 2009), but rather that TPJ is a hub within larger hierarchical networks that determine the context appropriate representation of sensory input (e.g., to update actions or expectations). In this sense, the specific function of TPJ is defined within the context of a network of regions. For example, TPJ is sometimes co-activated with the default mode network (DMN), but sometimes also with task-related networks serving perceptual, somatosensory, auditory processing and motor outputs using different effectors (Friedrich et al., 1998; Downar et al., 2002; Fox et al., 2005; Serences et al., 2005; Anticevic et al., 2010; Mars et al., 2012; Bzdok et al., 2013). For example, the appearance of a stimulus that predicted the onset of a target that required a right-handed response, produced increased functional connectivity between right TPJ and bilateral dorsal attentional control regions such as FEF and IPS and left motor cortex (Geng and Mangun, 2011). In another task, a right TPJ region showed increased connectivity with somatosensory cortex contralateral to a touched hand and visual cortex when subjects experienced incongruent multisensory events (Silani et al., in press).

**Fig. 3:**
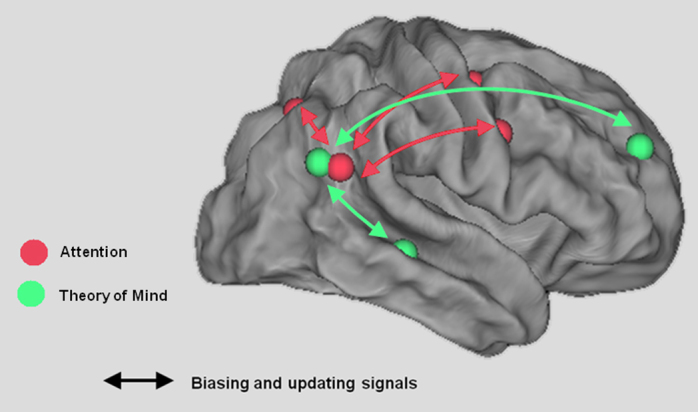
Schematic illustration of TPJ as a context updating hub in different domain-specific cortical networks. The maps are depicted on the flattened brain surface of the PALS atlas as implemented in Caret 5.65 (Van Essen, 2005). Coordinates for the red (attention-related) spheres were derived from a study by Serences et al. (2005). Coordinates for the green (TOM-related) activations were taken from Gweon et al. (2012).

The flexible integration between sensory, motor, and decisional regions can also be illustrated between domains. Fig. 3 provides a schematic illustration of how activations typically observed in attention and theory of mind may contribute to function-specific networks. The illustration depicts the attentional and ToM hubs as being overlapping anatomical neighbors, but the question of whether these functional modules are anatomically overlapping or distinct is still debated (e.g., see Fig. 1). More importantly, Fig. 3 depicts the hypothesis that there is context-specific coupling between TPJ (or subregions within TPJ) and other domain-specific brain regions that create task-specific networks. While TPJ may be composed of functionally distinct subparts (Bzdok et al., 2013), we would suggest that these regions are engaged in similar computations to update internal models in different behavioral and cognitive contexts. The possibility that the exact function of TPJ is itself defined by the context of the current task is intriguing and suggests that the context (which may be updated) involves the specific pattern of coupling between regions during a task (DiQuattro and Geng, 2011).

Although there are still many details that need to be specified with regard to the contextual updating hypothesis, the framework is a significant deviation from the current theories and therefore stands as a reasonable alternative hypothesis: instead of initiating “bottom-up” attentional reorienting, we hypothesize that TPJ has a more general post-perceptual function in evaluating and integrating stimulus information with internal models of task performance and expectations. We note that the contextual updating hypothesis does not leave attentional reorienting without a mechanism. It is reasonable that unattended, but relevant stimuli be assigned attentional priority through “dorsal attentional” network regions in a manner similar to other salient or relevant stimuli (Treue and Martinez Trujillo, 1999; Schall, 2004; Goldberg et al., 2006; Gottlieb, 2007; Lovejoy and Krauzlis, 2010). More studies, perhaps using tasks in which the context changes over time, are needed to assess the two main predictions: that task-defined responses and violations of expectations will produce the largest responses in TPJ.

## 5 Relationship of contextual updating to TPJ in other domains

Any theory of TPJ function must go beyond any one sub-discipline in order to account for its involvement in different cognitive processes. The contextual updating hypothesis is appealing because similar ideas have been suggested in many domains. For example, it has been proposed that right TPJ encodes an internal model of the body and engages in a “test-for-fit” process to determine whether sensory events belong to one's own body (Tsakiris et al., 2008). This hypothesis was supported by the finding that TMS over right TPJ interfered with the integration of multisensory information into the representation of one's own body. Similarly, out-of-body experiences based on the incorporation of visual and tactile information have been associated with bilateral TPJ using fMRI and voxel-based lesion symptom mapping analyses (Ionta et al., 2011). Although these data are in relation to a very different phenomena than attentional capture, they are similar in that they both involve the updating of an internal mental model (e.g., of one's own body, where to attend, or what action to take) by sensory events relevant to the current task.

Similarly, the contextual updating hypothesis helps to reconcile the debate between TPJ as a cognitive module for theory-of-mind (ToM) and attention tasks by suggesting a common computation occurs in both situations. The contextual updating hypothesis specifically refers to the TPJ activation and does not suggest that attentional reorienting itself does not occur during ToM (i.e., attentional reorienting may be involved in ToM tasks). While there may be anatomically separate functional modules in the posterior parietal cortex that map onto existing cognitive domains (Scholz et al., 2009), we argue that there is utility in considering contextual updating as a common computation in a variety of tasks that selectively activate TPJ. For example, in the Sally-Anne task (Baron-Cohen et al., 1985), the key to ToM is the ability to infer what is in the mind of Sally, whose marble has been moved by Anne while she was out of the room. In order to correctly indicate that Sally will search for the marble where she left it (and not where the observer knows it to be), the observer must update his/her internal model of where the marble currently is to reflect the context when Sally left the room (Saxe and Kanwisher, 2003; Decety and Lamm, 2007a; Perner and Aichhorn, 2008; Mar, 2011; Gweon et al., 2012). Thus, the activation of TPJ during ToM may reflect an update in contextual representation that is necessary to take another person's perspective, but may not be the perspective taking, per se. Similar computations are involved in other ToM tasks that require subjects to encode stories, picture sequences, and games that require the ability to attribute thoughts, beliefs, and other mental states to another person (Saxe and Kanwisher, 2003; Saxe, 2006; Mar, 2011). Such a perspective avoids the incompatibility of interpreting TPJ activations as strictly attentional reorienting or for theory-of-mind (Decety and Lamm, 2007a; Hein and Knight, 2008; Mitchell, 2008; Perner and Aichhorn, 2008; Greene et al., 2009; Scholz et al., 2009; Young et al., 2010).

Although a comprehensive review of other cognitive domains is beyond the scope of this current review, we note that the idea of contextual updating would be compatible with a number of other studies showing TPJ involvement in altruism (Morishima et al., 2012) and empathy (Morelli et al., 2013), perspective taking and imitation (Santiesteban et al., 2012), processing lies (Behrens et al., 2008) and evaluating the emotional states of oneself relative to another (Silani et al., in press). In all these studies, an internal schema or model of environmental stimuli or another person's behaviors or thoughts must be updated relative to one's own beliefs, thoughts or actions within the same context. Interestingly, when there is no need to update potentially conflicting representations of another person's beliefs relative one's own beliefs or behavior, right TPJ is no longer involved in attributing mental states to others (Santiesteban et al., 2012).

The broader characterization of TPJ in contextual updating is consistent with its role in attentional and social-cognitive domains. Although these situations are superficially very different, they all involve a stimulus-triggered event update of internal mental models of the external environment (or person), which are then used to predict future outcomes (i.e., one's own behaviors, another person's behaviors or beliefs, or stimulus occurrences). A challenge for future studies of TPJ will be to provide insight into how the general function of contextual updating is made specific for a highly variable range of tasks and cognitive domains (e.g., through learning).

## 6 Anatomy

A critical aspect of the discussion about TPJ relates to its anatomical location. Thus far, we have used the term TPJ loosely to refer to a cluster of regions in the inferior parietal lobule extending into the superior temporal gyrus (see Figs. 1 and 2). One possible reason for the variability in location is that TPJ is typically defined in fMRI through functional localization in an individual or an experiment-wide group analysis. The identification of functional regions in this manner is common practice in much of the human neuroimaging literature and is used routinely to define perceptual areas with boundaries that vary from individual-to-individual (Sereno et al., 1995; Kanwisher et al., 1997; Gandhi et al., 1999). However, localization of TPJ in humans is complicated by a lack of standard anatomical or functional definitions. Additionally, there is controversy over the existence of a non-human primate homologoue, which precludes use of monkey models to constrain the human work (Constantinidis and Steinmetz, 2001; Grefkes and Fink, 2005; Caspers et al., 2006; Orban et al., 2006; Rushworth et al., 2006; Husain and Nachev, 2007; Uddin et al., 2010; Oleksiak et al., 2011; Thiebaut de Schotten et al., 2011).

Nevertheless, there are general conventions and TPJ is typically described as being near “the posterior sector of the superior temporal sulcus (STS) and gyrus (STG) and the ventral part of the supramarginal gyrus (SMG)” p. 307 (Corbetta et al., 2008) (cf. Fig. 1). More precise definitions have been offered (Mort et al., 2003; Chambers et al., 2004b), but have not been systematically applied in the literature. The consequence of this non-uniformity is that the coordinates of TPJ can vary considerably between studies within the region of the inferior parietal lobe, including the inferior supramarginal gyrus, angular gyrus, and/or posterior portions of the superior temporal sulcus and gyrus. Although these differences can be seemingly small from study-to-study, they have played a critical role in the debate regarding functional specialization (Decety and Lamm, 2007a; Mitchell, 2008; Scholz et al., 2009; Kubit and Jack, 2013). In this review, we did not select studies based on the reported anatomical location, but rather included as many as we could that self-identified as TPJ (see Fig. 1).

One exciting recent development has been creation of a new cytoarchitectonic maps based on postmortem human brains (Caspers et al., 2006, 2008; Amunts et al., 2007; Devlin and Poldrack, 2007; Wang et al., 2012). Whereas Brodmann's areas 40 and 39 corresponded to the supramarginal and angular gyrus, respectively, Caspers and colleagues have now subdivided the inferior parietal lobe into seven areas (see Fig. 2B). These include five in BA 40 and two in BA 39. Moreover, the individual brains from the cytoarchitectonic parcellation of the inferior parietal lobe have been normalized into stereotaxic space to generate maximum probability maps for each cortical subdivision. It is now possible to assess the likelihood of any functional or structural result being in each cortical subarea using these maps, which will significantly facilitate more precise descriptions of TPJ anatomy despite interindividual variability in microanatomy and a lack of macroanatomical markers (Caspers et al., 2008). Several studies have already begun to use these maps to establish differences in connectivity and precise functional localization using diffusion tensor imaging (DTI) and resting state functional connectivity (rs-fcMRI) analyses (Cohen et al., 2008; Uddin et al., 2010; Mars et al., 2011, 2012). These studies have shown specialization in connectivity for the newly defined subareas, much of which is consistent with previous findings that regions of the posterior parietal cortex have highly diverse connections with medial temporal lobe regions, frontal and temporal cortex (Mesulam, 1990).

Interestingly, studies that have used the cytoarchtectonic parcelation scheme to identify fMRI activations in TPJ, have been more mixed. For example, in a cued attention task, invalid targets activated both PGa and PFm, regions in the anterior portion of the angular gyrus and posterior supramarginal gyrus (Gillebert et al., 2012). This is consistent overall with the characterization of TPJ as being at the intersection of the two gyri bordering the superior temporal sulcus (Corbetta and Shulman, 2002; Husain and Nachev, 2007), but also suggests that the mapping between anatomical divisions and functional localization is imperfect. This can be due to the insufficient spatial resolution of fMRI, imprecision in tasks relative to the computation of that region, interindividual variability and the application of spatial normalization techniques, or to the fact that higher-order cognitive activity may not subdivide cleanly along anatomical borders (Gillebert et al., 2012). This work suggests that understanding the role of each subdivision in cognition may require convergent methodologies that can isolate the differences in anatomy, local functional representations, stable anatomical connections, as well as potentially more flexible functional network connectivity. Nevertheless, the new anatomical divisions between regions of the inferior parietal cortex and potentially the temporal lobe, will aid finer classification of imaging results and targets for brain stimulation.

## 7 Summary

Regions of the inferior parietal lobe within the supramarginal and angular gyri have commonly been referred to collectively as the temporoparietal junction. In the extant literature on attention, the exact location of TPJ has varied between studies considerably since the definition is frequently based on functional contrasts across a group rather than individual functional or anatomical locations. It is possible that different studies are actually referring to different subregions that are near anatomical neighbors. Although it may well be the case that the results we reviewed will someday be discovered to be anatomically distinct, the review was aimed at addressing the question of what common computation the results attributed to TPJ might perform; even if the specific nature of representations between subregions are separable, neighbors are likely to be computationally similar.

Here we have proposed that a likely framework for TPJ function can be found in the idea of contextual updating: that the purpose of TPJ is to update internal models of the environment (including other people) for the purpose of constructing appropriate expectations and responses. These may occur for attention to simple stimuli as well as for understanding social cognition and our bodies. The anatomical connectivity between TPJ and the medial temporal lobe and frontal regions situates it well to integrate internal (or memory) representations of the current context with context-appropriate sensory-to-motor transformations. Such a framework describes well the myriad deficits that are involved with brain damage to this region and the many different tasks that produce activation in this region, but there is still much to be done to characterize the functional profile of TPJ as well its anatomical demarcations. Nevertheless, we hypothesize that the context of current events and behavioral goals will be critical to the function of this region. Much is still to be discovered about the function of TPJ, but the evidence indicates that we need to reevaluate the idea that it is involved in sending a fast-latency interrupt signal to dorsal attentional control regions and that its function is right-lateralized.

## Figures and Tables

**Table 1:** The studies were selected from the meta-analytic work by Decety and Lamm (2007a,b) and updated by the studies reviewed in Kubit and Jack (2013) as well as results from Pubmed literature search. ATTENTION.

Study	TAL		
	*x*	*y*	*z*
Asplund CL, Todd JJ, Snyder AP, Marois R. 2010. A central role for the lateral prefrontal cortex in goal-directed and stimulus-driven attention. Nat Neurosci 13:507–512.	46	−56	27
Arrington C, Carr T, Mayer A, Rao S. 2000. Neural mechanisms of visual attention – object-based selection of a region in space. J Cogn Neurosci 12(S2):106–17.	47	−62	26
Astafiev SV, Shulman GL, Corbetta M. 2006. Visuospatial reorienting signals in the human temporo-parietal junction are independent of response selection. Eur J Neurosci 23:591–96.	51	−51	26
Braver TS, Barch DM, Gray JR, Molfese DL, Snyder A. 2001. Anterior cingulate cortex response conflict: effects of frequency, inhibition and errors. Cereb. Cortex 11:825–36.	56	−48	24
Chen Q, Weidner R, Vossel S, Weiss PH, Fink GR. 2012. Neural mechanisms of attentional reorienting in three-dimensional space. J Neurosci 32:13352–62.	51	−40	16
Corbetta M, Kincade JM, Ollinger JM, McAvoy MP, Shulman GL. 2000. Voluntary orienting is dissociated from target detection in human posterior parietal cortex. Nat Neurosci 3:292–7.	53	−49	30
	57	−45	12
	39	−47	48
Corbetta M, Kincade JM, Shulman GL. 2002. Neural Attention systems for visual orienting and their relationships to spatial working memory. J Cogn Neurosci 14:508–23.	57	−45	12
DiQuattro NE, Sawaki R, Geng JJ. in press. Effective connectivity during feature-based attentional capture: Evidence against the attentional reorienting hypothesis of TPJ. Cereb. Cortex	50	−38	20
Doricchi F, Macci E, Silvetti M, Macaluso E. 2010. Neural correlates of the spatial and expectancy components of endogenous and stimulus-driven orienting of attention in the Posner task. Cereb. Cortex 20:1574–1585.	60	−46	28
Downar J, Crawley AP, Mikulis DJ, Davis KD. 2000. A multimodal cortical network for the detection of changes in the sensory environment. Nat Neurosci 3:277–83.	53	−40	16
Downar J, Crawley AP, Mikulis DJ, Davis KD. 2001. The effect of task relevance on the cortical response to changes in visual and auditory stimuli: an event-related fMRI study. NeuroImage 14:1256–67.	58	−43	17
	57	−48	10
Downar J, Crawley AP, Mikulis DJ, Davis KD. 2002. A cortical network sensitive to stimulus salience in a neutral behavioral context across multiple sensory modalities. J Neurophysiol 87:615–20.	56	−36	24
	56	−36	24
	55	−53	4
Geng JJ, Mangun GR. 2011. Right temporoparietal junction activation by a salient contextual cue facilitates target discrimination. NeuroImage 54:594–601.	46	−55	17
Giessing C, Thiel CM, Roesler F, Fink G. 2006. The modulatory effects of nicotine on parietal cortex activity in a cued target detection task depend on cue reliability. Neuroscience 137:853–64.	44	−46	19
Indovina I, Macaluso E. 2007. Dissociation of stimulus relevance and saliency factors during shifts of	50	−36	28
visuospatial attention. Cereb. Cortex 17:1701–11.			
Kincade M, Abrams RA, Astafiev SV, Shulman GL, Corbetta M. 2005. An event-related functional magnetic resonance imaging study of voluntary and stimulus-driven orienting of attention. J Neurosci 25:4593–604.	50	−48	26
	51	−51	26
	54	−48	30
Konrad K, Neufang S, Thiel CM, Specht K, Hanisch C, Fan J, and others. 2005. Development of attentional networks: an fMRI study with children and adults. NeuroImage 28:429–39.	58	−38	14
Lepsien J, Pollmann S. 2006. Covert reorienting and inhibition of return: an event-related fMRI study. J Cogn Neurosci 14:127–44.	56	−52	16
	55	−49	15
Macaluso E, Frith CD, Driver J. 2002. Supramodal effects of covert spatial orienting triggered by visual or tactile events. J Cogn Neurosci 143:389–401.	60	−48	32
Mattler U, Wuestenberg T, Heinze H-J. 2006. Common modules for processing invalidly cued events in the human cortex. Brain Res 1109:128–141.	59	−50	6
Mayer AR, Dorflinger JM, Rao SM, Seidenberg M. 2004. Neural networks underlying endogenous fMRI and exogenous visual-spatial orienting. NeuroImage 23:534–41.	54	−51	28
	55	−53	27
Mayer AR, Franco AR, Harrington D L. 2009. Neuronal modulation of auditory attention by informative and uninformative spatial cues. Hum Brain Mapp 30:1652–1666.	57	−47	26
	55	−48	8
Mayer AR, Harrington D, Adair JC, Lee R. 2006. The neural networks underlying endogenous auditory covert orienting and reorienting. NeuroImage 30:938–949.	54	−45	8
Mitchell JP. 2008. Activity in right temporo-parietal junction is not selective for theory-of-mind. Cereb. Cortex 18:262–271.	59	−45	27
Natale E, Marzi CA, Macaluso E. 2009. FMRI correlates of visuo-spatial reorienting investigated with an attention shifting double-cue paradigm. Hum Brain Mapp 30:2367–2381.	59	−46	15
Ruff CC, Driver J. 2006. Attentional preparation for a laterilized visual distractor: behavioral and fMRI evidence. J Cogn Neurosci 18:522–38.	56	−36	16
Scholz J, Triantafyllou C, Whitfield-Gabrieli S, Brown EN, Saxe R. 2009. Distinct regions of right temporo-parietal junction are selective for theory of mind and exogenous attention. PLoS One 4:e4869.	57	−58	41
Serences JT, Shomstein S, Leber AB, Golay X, Egeth HE, Yantis S. 2005. Coordination of voluntary and stimulus-driven attentional control in human cortex. Psychol Sci 16:114–122.	55	−44	24
Shulman GL, McAvoy MP, Cowan MC, Astafiev SV, Tansy AP, d’Avossa G, Corbetta M. 2003. Quantitative analysis of attention and detection signals during visual search. J Neurophysiol 90:3384–97.	51	−49	28
	45	−49	42
	53	−39	40
Shulman GL, Astafiev S V, Franke D, Pope DLW, Snyder AZ, McAvoy MP, Corbetta M. 2009. Interaction of stimulus-driven reorienting and expectation in ventral and dorsal frontoparietal and basal ganglia-cortical networks. The Journal of Neuroscience 29:4392–4407.	52	−49	17
Shulman GL, Pope DL, Astafiev S V, McAvoy MP, Snyder AZ, Corbetta M. 2010. Right hemisphere dominance during spatial selective attention and target detection occurs outside the dorsal frontoparietal network. J Neurosci 30:3640–51.	52	−8	14
	46	−45	26
Thiel CM, Zilles K, Fink GR. 2004. Cerebral correlates of alerting, orienting and reorienting of visuospatial attention: an event-related fMRI study. NeuroImage 21:318–28.	45	−66	17
Todd JJ, Fougnie D, Marois R. 2005. Visual short-term memory load suppresses temporo-parietal junction activity and induces inattentional blindness. Psychol Sci 16:965–72.	59	−47	24
Vossel S, Thiel CM, Fink GR. 2006. Cue validity modulates the neural correlates of covert endogenous orienting of attention in parietal and frontal cortex. NeuroImage 32:1257–64.	56	−55	17
Vossel S, Weidner R, Driver J, Friston KJ, Fink GR. 2012. Deconstructing the architecture of dorsal and ventral attention systems with dynamic causal modeling. J Neurosci 32:10637–48.	58	−57	18
Vossel S, Weidner R, Thiel CM, Fink GR. 2009. What is ‘odd’ in Posner's location-cueing paradigm? Neural responses to unexpected location and feature changes compared. J Cogn Neurosci 21:30–41.	65	−42	12

THEORY OF MIND			
Study	TAL		
	*x*	*y*	*z*
Abraham A, Rakoczy H, Werning M, von Cramon DY, Schubotz RI. 2010. Matching mind to world and vice versa: Functional dissociations between belief and desire mental state processing. Soc Neurosci 5:1–18.	57	−36	21
Aichorn M, Perner J, Weiss B, Kronbichler M, Staffen W, Ladurner G. 2009. Temporo-parietal junction activity in theory-of-mind tasks: falseness, beliefs, or attention. J Cogn Neurosci 21:1179–92.	56	−52	16
	53	−49	19
Baron-Cohen S, Ring HA, Wheelwright S, Bullmore ET, Brammer MJ, Simmons, A, Williams SCR. 1999. Social intelligence in the normal and autistic brain: an fMRI study. Eur J Neurosci 11:1891–8.	40	−58	20
Brunet E, Sarfati Y, Hardy-Bayle MC, Decety J. 2000. A PET investigation of attribution of intentions to others with a non-verbal task. NeuroImage 11:157–66.	58	−62	22
Brunet E, Sarfati Y, Hardy-Baylé MC, Decety J. 2003. A PET study of the attribution of intentions to others in schizophrenia. Neuropsychologia 41:1574–82.	55	−50	19
Castelli F, Happe F, Frith U, Frith CD. 2000. Movement and mind: a functional imaging study of perception and interpretation of complex intentional movement patterns. NeuroImage 12:314–25.	60	−56	12
Decety J, Jackson PL, Sommerville JA, Chaminade T, Meltzoff AN. 2004. The neural bases of cooperation and competition: an fMRI study. NeuroImage 23:744–51.	51	−44	45
Den Ouden HEM, Frith U, Frith CD, Blakemore S-J. 2005. Thinking about intentions. NeuroImage 28:787–96.	48	−66	39
Dohnel K, Schuwerk T, Meinhardt J, Sodian B, Hajak G, Sommer M. 2012. Functional activity of the right temporoparietal junction and of the medial prefrontal cortex associated with true and false belief reasoning. NeuroImage 60:1652–1661.	49	−65	10
	51	−43	14
	57	−38	4
Fletcher PC, Happe F, Frith U, Baker SC, Dolan RJ, Frackowiak RSJ, Frith CD. 1995. Other minds in the brain: a functional imaging study of theory of mind in story comprehension. Cognition 57:109–28.	42	−50	24
Gallagher HL, Happe F, Brunswick N, Fletcher PC, Frith U, Frith CD. 2000. Reading the mind in cartoons and stories: an fMRI study of theory of mind in verbal and nonverbal tasks. Neuropsychologia 38:1–21.	46	−56	26
Grezes J, Frith CD, Passingham RE. 2004. Inferring false beliefs from the actions of oneself and others: an fMRI study. NeuroImage 21:744–50.	42	−59	27
Hartwright CE, Apperly IA, Hansen PC. 2012. Multiple roles for executive control in belief-desire reasoning: distinct neural networks are recruited for self perspective inhibition and complexity of reasoning. NeuroImage 61: 921–930.	51	−51	25
Hynes CA, Baird AA, Grafton ST. 2006. Differential role of the orbitofrontal lobe in emotional versus cognitive perspective-taking. Neuropsychologia 44:374–83.	53	−51	19
Jenkins AC, Mitchell JP. 2010. Mentalizing under uncertainty: dissociated neural responses to ambiguous and unambiguous mental state inferences. Cereb. Cortex 20:404–410.	53	−53	23
Kobayashi C, Glover GH, Temple E. 2006. Cultural and linguistic influence on neural bases of ‘Theory of Mind’: an fMRI study with Japanese bilinguals. Brain Lang 98:210–220.	50	−40	19
Kobayashi C, Glover GH, Temple E. 2008. Switching language switches mind: linguistic effects on developmental neural bases of ‘Theory of Mind’. Soc Cogn Affect Neurosci 3:62–70.	51	−42	21
Mitchell JP. 2008. Activity in right temporo-parietal junction is not selective for theory-of-mind. Cereb. Cortex 18:262–271.	53	−48	27
Moriguchi Y, Ohnishi T, Lane RD, Maeda M, Mori T, Nemoto K, and others. 2006. Impaired of self-awareness and theory of mind: an fMRI study mentalizing in alexithymia. NeuroImage 32:1472–82.	52	−46	14
Ohnishi T, Moriguchi Y, Matsuda H, Mori T, Hirakata M, Imabayashi E, and others. 2004. The neural network for the mirror system and mentalizing in normally developed children: an fMRI study. NeuroReport 15:1483–8.	48	−42	19
Perner J, Aichhorn M, Kronbichler M, Staffen W, Ladurner G. 2006. Thinking of mental and other representations: the roles of left and right temporo-parietal junction. Soc Neurosci 1:245–58.	53	−54	28
	48	−52	34
Rilling JK, Sanfey AG, Aronson JA, Nystrom LE, Cohen JD. 2004. Opposing BOLD responses to reciprocated and unreciprocated altruism in putative reward pathways. NeuroReport 15:2539–43.	42	−52	16
Rilling JK, Sanfey AG, Aronson JA, Nystrom LE, Cohen JD. 2004. The neural correlates of theory of mind within interpersonal interactions. NeuroImage 22:1694–703.	48	−55	27
	40	−55	32
Ruby P, Decety J. 2003. What you believe versus what you think they believe? A neuroimaging study of conceptual perspective taking. Eur J Neurosci 17:2475–80.	44	−66	36
Samson AC, Zysset S, Huber O. 2008. Cognitive humor processing: different logical mechanisms in nonverbal cartoons-an fMRI study. Soc Neurosci 3:125–140.	31	−76	33
Saxe R, Kanwisher N. 2003. People thinking about people—the role of the temporo-parietal junction in theory of mind. NeuroImage 19:1835–42.	50	−55	28
Saxe R, Powell LJ. 2006. It's the thought that counts: specific brain regions for one component of theory of mind. Psychol Sci 17:692–9.	52	−52	18
Saxe R, Schulz LE, Jiang YV. 2006. Reading minds versus following rules: dissociating theory of mind and executive control in the brain. Soc Neurosci 1:284–98.	56	−54	19
Saxe R, Wexler A. 2005. Making sense of another mind: the role of the right temporo-parietal junction. Neuropsychologia 43:1391–9.	53	−51	25
Scholz J, Triantafyllou C, Whitfield-Gabrieli S, Brown EN, Saxe R. 2009. Distinct regions of right temporo-parietal junction are selective for theory of mind and exogenous attention. PLoS One 4:e4869.	59	−53	32
Singer T, Kiebel SJ, Winston JS, Dolan RJ, Frith CD. 2004. Brain responses to the acquired moral status of faces. Neuron 41:653–62.	53	−46	19
Voellm BA, Taylor AN, Richardson P, Corcoran R, Stirling J, Mckie S, and others. 2006. Neuronal correlates of theory of mind and empathy: a functional magnetic resonance imaging study in nonverbal task. NeuroImage 29:90–8.	44	−75	20
Vogeley K, Bussfeld P, Newen A, Herrmann S, Happe F, Falkai P, and others. 2001. Mind reading: neural mechanism of theory of mind and self-perspective. NeuroImage 14:170–81.	58	−56	12
Walter H, Adenzato M, Ciaramidaro A, Enrici I, Pia L, Bara BG. 2006. Understanding intentions in social interaction: the role of the anterior paracingulate cortex. J Cogn Neurosci 16:1854–63.	56	−49	13
	50	−40	13
	58	−56	16
Young L, Dodell-Feder D, Saxe R. 2010. What gets the attention of the temporo-parietal junction? An fMRI investigation of attention and theory of mind. Neuropsychologia 48:2658–2664.	61	−53	23

EMPATHY			
Study	TAL		
	*x*	*y*	*z*
Botvinick M, Jha AP, Bylsma LM, Fabian SA, Solomon PE, Prkachin KM. 2005. Viewing facial expression of pain engages cortical areas involved in the direct experience of pain. NeuroImage 25:312–9.	68	−40	16
Decety J, Chaminade T. 2003. Neural correlates of feeling sympathy. Neuropsychologia 41:127–38.	59	−45	35
Hooker CI, Verosky SC, Germine LT, Knight RT, D’Esposito M. 2010 Mentalizing abouy emotion and its relationship to empathy. Soc Cogn Affect Neurosci, 3:2014–17.	46	−62	33
Hynes CA, Baird AA, Grafton ST. 2006. Differential role of the orbitofrontal lobe in emotional versus cognitive perspective-taking. Neuropsychologia 44:374–83.	53	−51	19
Jackson PL, Brunet E, Meltzoff AN, Decety J. 2006. Empathy examined through the neural mechanisms involved in imagining how I feel versus how you feel pain: an event-related fMRI study. Neuropsychologia 44:752–61.	48	−54	28
Jackson PL, Meltzoff AN, Decety J. 2005. How do we perceive the pain of others: a window into the neural processes involved in empathy. NeuroImage 24:771–9.	40	−47	39
Lamm C, Batson CD, Decety J. 2007. The neural basis of human empathy-effects of perspective-taking and cognitive appraisal. J Cogn Neurosci 19:1–7.	48	−60	44
Lawrence EJ, Shaw P, Giampietro VP, Surguladze S, Brammer MJ, David AS. 2006. The role of ‘shared representations’ in social perception and empathy: an fMRI study. NeuroImage 29:1173–84.	47	−45	41
Morelli, SA, Lieberman MD. 2013. The role of automaticity and attention in neural processes underlying empathy for happiness, sadness, and anxiety. Front Hum Neurosci 7:160.	50	−38	11
Moriguchi Y, Decety J, Ohnishi T, Maeda M, Mori T, Nemoto K, and others. 2007. Empathy and judging other's pain: an fMRI study of alexythymia. Cereb. Cortex 17:2223–34.	63	−33	35
Ruby P, Decety J. 2004. How would you feel versus how do you think she would feel? A neuroimaging study of perspective taking with social emotions. J Cogn Neurosci 16:988–99.	59	−53	23
Singer T, Seymour B, O’Doherty J, Kaube H, Dolan RJ, Frith CD. 2004. Empathy for pain involves the affective but not the sensory components of pain. Science 303:1157–61.	50	−49	13
Voellm BA, Taylor AN, Richardson P, Corcoran R, Stirling J, Mckie S, and others. 2006. Neuronal correlates of theory of mind and empathy: a functional magnetic resonance imaging study in nonverbal task. NeuroImage 29:90–8.	52	−57	19
